# Combination Hyaluronidase and Triamcinolone Acetonide Enzymatic Injections for Treatment of Ledderhose Disease: A Novel Technique and Case Series

**DOI:** 10.3390/clinpract16020035

**Published:** 2026-02-06

**Authors:** Paul Carroll, Alyson Boudreau, Haoning Hu, Ryan P. Lin, Bilal Louzati, Eddie Davis

**Affiliations:** MedStar Health, 9105 Franklin Square Drive Suite 214, Baltimore, MD 21237, USA; alyson.boudreau@medstar.net (A.B.); ryplin@gmail.com (R.P.L.); bilal.louzati@medstar.net (B.L.);

**Keywords:** hyaluronidase, Ledderhose Disease, enzymatic injections, plantar fibromatosis, fibroproliferative disorder, fibrotic tissue modulation

## Abstract

**Background:** Ledderhose Disease, or plantar fibromatosis, is a fibroproliferative disorder affecting the plantar fascia with limited effective treatment options. Although hyaluronidase has a long history of clinical use, it has not been previously used for Ledderhose Disease. This study explores the use of combined hyaluronidase and triamcinolone acetonide enzymatic injections as a novel and promising technique for managing Ledderhose Disease. **Methods:** This paper investigates the use of combination therapy with hyaluronidase, triamcinolone acetonide, and lidocaine injections in three patients with Ledderhose Disease. Injection protocols, dosage, frequency, and patient outcomes are all discussed. Additionally, this study explores the underlying mechanisms of hyaluronidase action in Ledderhose Disease, shedding light on its potential to modulate fibrotic tissue and alleviate symptoms. **Results:** All three patients treated with a series of hyaluronidase, triamcinolone acetonide, and lidocaine anesthetic injections experienced either a significant reduction in or elimination of nodules and associated pain within 6 weeks after initial injection. Patients were asymptomatic at two years follow-up after injections. **Conclusions:** The combination of hyaluronidase and triamcinolone acetonide injections significantly decreased pain and softened fibromas faster than triamcinolone acetonide injection alone, as explored in previous studies. Large prospective studies are needed to further compare enzymatic injection therapies in the management of Ledderhose Disease.

## 1. Introduction

Ledderhose Disease is characterized by the formation of nodules and fibrous tissue in the plantar fascia, resulting in pain, discomfort, and impaired mobility. Classified as a rare disease, it is estimated to affect fewer than 200,000 individuals in the United States [1]. Ledderhose Disease can be classified based on a tumor staging system, which assigns grades from I to IV based on focal or multifocal disease, adherence to skin, and/or extension to flexor sheath [2]. While various treatment modalities have been proposed due to its similarities to Peyronie’s and Dupuytren’s diseases, the absence of a definitive and universally effective approach has spurred the search for innovative interventions [3]. Such documented treatments include steroid injections, verapamil, imatinib, radiation therapy, extracorporeal shock wave therapy, tamoxifen, sorafenib, mitomycin C, and collagenase, with varying degrees of effectiveness [4]. Triamcinolone acetonide injections have been used in the treatment of Ledderhose Disease with some success [5]. Hyaluronidase, known for its ability to hydrolyze hyaluronic acid, presents a promising additive affect to modulate fibrotic tissue [6].

Hyaluronan (known as hyaluronic acid [HA]) is a major glycosaminoglycan of the extracellular matrix (ECM) whose synthesis and pericellular assembly are upregulated in response to transforming growth factor-β1 (TGF-β1) [7,8]. HA facilitates TGF-β1-dependent signaling through CD44 and epidermal growth factor receptor (EGFR), supporting both proliferation and differentiation of fibroblasts to myofibroblasts [9,10]. Myofibroblast-differentiated fibroblasts, as observed in Ledderhose Disease, are responsible for the excessive deposition of ECM. Unchecked myofibroblast proliferation leads to the accumulation of dense fibrotic tissue and the formation of hard nodules [8,11]. Hyaluronidase, which has been used in the United States for over 60 years, was initially developed to spread or diffuse a substance by increasing connective tissue permeability by hydrolysis of HA [12]. Hyaluronidase is an endoglycosidase that breaks down hyaluronic acid into monosaccharides by cleaving glycosidic bonds and, to some extent, break down other acid mucopolysaccharides in the connective tissue [12]. The current literature also supports its role as a modulator of ECM components, providing a rationale for its use in disorders involving fibrosis [12].

The use of combination enzymatic injections is not a new concept and has been employed for years in the management of other fibroblastic disorders throughout the body [9]. The combination of betamethasone, hyaluronidase, and lidocaine has been investigated for the treatment of Peyronie’s disease [13]. In the 1960s and 1970s, enzymatic fasciotomy using trypsin, alpha-chymotrypsin, hyaluronidase, and thiomucas for the treatment of Dupuytren’s contracture was also explored with success [14,15,16,17].

Recent studies emphasize the efficacy of enzymatic injections in enhancing the diffusion of therapeutic agents into targeted tissues [3,7]. This localized, targeted approach holds promise for addressing the fibrotic nodules characteristic of Ledderhose Disease. Emerging evidence suggests that hyaluronidase injections may play a crucial role in symptom mitigation and functional improvement in individuals affected by musculoskeletal fibrosis [8]. This study focuses on the application of hyaluronidase and triamcinolone acetonide enzymatic injections as a potential therapeutic option.

## 2. Injection Technique

**Figure 1:**
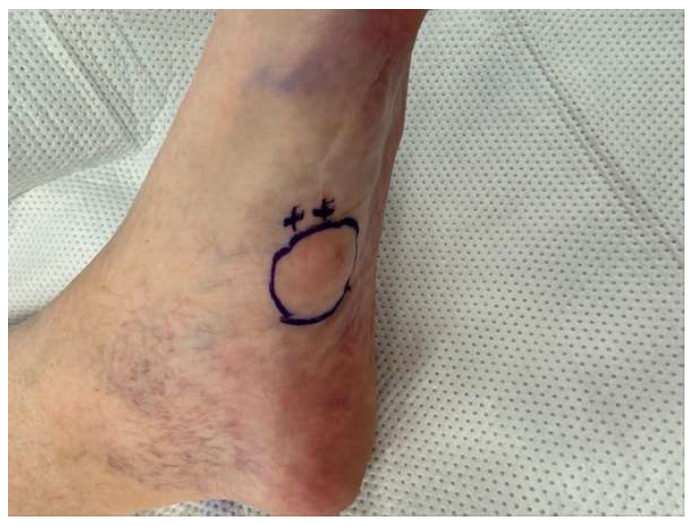
Identification and marking of injection site for lidocaine with epinephrine and enzymatic injection.

**Figure 2:**
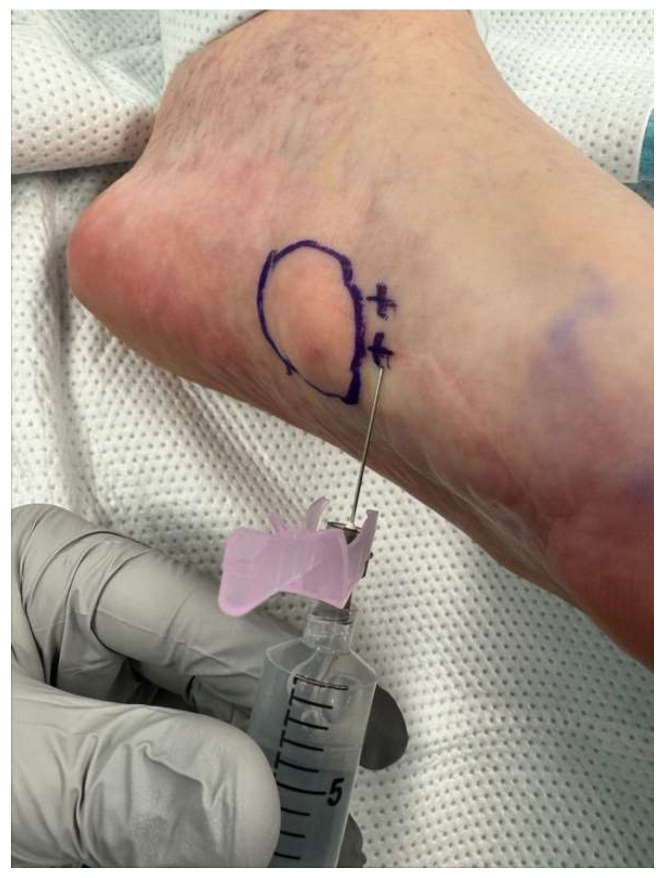
Lidocaine 1% with epinephrine is infiltrated around fibroma to reduce pain and bleeding during enzymatic injections.

**Figure 3:**
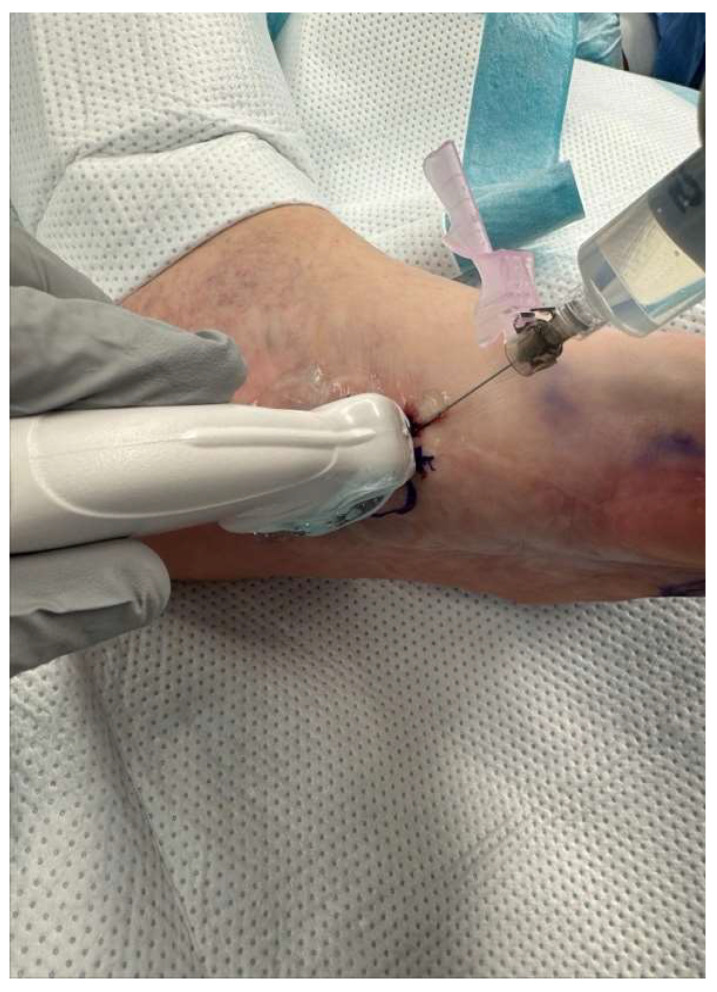
Ultrasound-guided injection of hyaluronidase, triamcinolone acetonide, and lidocaine 1% plain into fibroma.

The technique for administering hyaluronidase and triamcinolone acetonide enzymatic injections was developed by the senior author (E.D.). The injection technique is described below:

1. The patient sits supine in the exam chair, with feet placed on the footrest. The fibrotic nodules within the plantar fascia are identified through palpation or guided by imaging techniques such as ultrasound. The injection sites are marked with a marking pen based on the location and distribution of nodules (Figure 1).
2. The injection site is prepared by disinfecting the plantar surface, typically using an antiseptic solution.
3. Ethyl chloride spray is recommended to anesthetize the skin prior to injection. Approximately 3–5 milliliters (mL) of lidocaine 1% or 2% with epinephrine 1:100,000 is infiltrated around the lesion with a 25-gauge, 1-1/2” needle. The needle is inserted at the marked sites, aiming to reach the fibrotic nodules within the plantar fascia (Figure 2).
4. For each fibroma, using the sterile technique, a recommended combination of 1 mL of hyaluronidase (150 international units [IU]), 1 mL of lidocaine 1% plain or local anesthetic of choice, and 0.25 mL of triamcinolone acetonide (Kenalog-40) is prepared in a 3 mL syringe with a 22-gauge, 1-1/2” needle.
5. The fibroma is visualized under ultrasound (performing the injection with ultrasound is recommended to increase accuracy). The prepared hyaluronidase solution is injected into the targeted nodules at the marked sites. The fibroma is infiltrated with approximately half of the solution into the center of the lesion, with the remaining solution infiltrated around the fibroma. Care is taken to ensure a slow and controlled injection, allowing for optimal diffusion of the enzymatic solution within the fibrous tissue. These steps are repeated for each fibroma (Figure 3).
6. Post-Injection Care: The patient is allowed to immediately bear weight in their desired footwear. The patient is informed that they may experience increased pain over the next few days.
7. Protocol: A series of three injections at three-week intervals should be considered. After the third set of injections, depending on the size and pain level of the fibroma, additional injections at three-week intervals may be continued. Once the fibroma is no longer symptomatic, it is recommended that patients start application of verapamil 15% transdermal gel, one to two times a day, indefinitely over each mass. For multiple lesions on one foot, a posterior tibial nerve block may be performed for better pain relief during injections.

## 3. Case Studies

Consent was obtained for all three patients for publication.

**Case Study 1:** A 49-year-old female with a medical history of alcoholism, hypertension, hyperlipidemia, prosthetic knee joint infection, and smoking presented with a 6-month history of a painful nodule on the plantar aspect of the left midfoot. The patient had not previously received treatment for the nodule. Examination demonstrated a painful, firm, non-mobile nodule measuring 0.64 cm^2^ on the medial band of the plantar fascia of the midfoot.

The patient underwent a series of four ultrasound-guided injections, each consisting of 150 IU hyaluronidase with 1 mL of 1% lidocaine plain and 0.25 mL triamcinolone acetonide. Each injection was administered three weeks apart, totaling a treatment duration of nine weeks. The patient reported improvement of symptoms three days after the first injection, experiencing a softening of the nodule and 50% pain reduction compared to before injections. She reported her pain level at its worst as 8/10 on visual analog scale (VAS), with no signs of ecchymosis. After each subsequent injection, the patient noted a reduction in both a 25% pain and nodule size. After the fourth injection, the nodule reduced in size to 0.04 cm^2^, and the patient was pain-free (0/10 VAS), opting to stop injections. The patient reported some dry skin around the injection site, which improved with the use of topical moisturizing cream. She declined to use the topical 15% verapamil gel daily for recurrence prevention. At the one-year and two-year follow-up, the nodule was undetectable, and the patient remained pain-free.

**Case Study 2:** A 59-year-old male presented with a 2-year history of painful masses on the plantar aspect of both feet. The pain had progressively worsened, particularly with weight-bearing, due to mass enlargement six months prior to the initial visit. The past medical history was non-contributory except for keloid formation. The patient was not taking any medications. Examination demonstrated two firm, tender masses affixed to the medial margin of the central band of the left plantar fascia and one firm, tender mass affixed to the distal aspect of the medial margin of the central band of the right plantar fascia.

Sonographic examination of the left foot demonstrated two hypoechoic masses with a disorganized fiber pattern characteristic of plantar fibroma, measuring 0.23 cm^2^ (#1) and 0.29 cm^2^ (#2). Sonography of the right foot demonstrated one hypoechoic mass with a disorganized fiber pattern characteristic of plantar fibroma affixed to the distal medial margin of the central band of the plantar fascia, measuring 1.51 cm^2^.

The patient was treated with a series of 3 intralesional injections utilizing 150 IU hyaluronidase, 0.25 mL triamcinolone acetonide, and 1 mL of 1% lidocaine at weeks 3 and 7. The patient reported 50% pain reduction after the first injection compared. Prior to injections, pain in all fibromas was 9/10 on the VAS. At week 7, sonography demonstrated that the fibromas on the left foot had decreased in size to 0.11 cm^2^ (#1) and 0.13 cm^2^ (#2), respectively, whereas the fibroma on the right foot had decreased in size to 0.89 cm^2^. The patient was pain-free (0/10 VAS) and began a regimen of topical verapamil 15% gel twice daily. At one and two years follow-up, the patient remained pain-free and fibroma size remained unchanged.

**Case Study 3:** A 65-year-old female with a past medical history of hypertension and bilateral Dupuytren’s contractures presented to the clinic with a 15-year history of recurrent fibromas on both feet. Previous treatment included simple excision of fibromas on the left foot, which recurred within one year after excision. The patient experienced long-term neuropathic pain following the excision of the fibroma with a 10/10 on VAS. She had also used verapamil cream regularly for both hands and feet with some relief.

Examination initially revealed three fibromas on the left foot and one fibroma on the right foot. The two plantar fibromas on the left foot were tender to palpation and affixed to the medial margin of the central band of the plantar fascia. A hypertrophic scar was present over the left medial longitudinal arch in proximity to the fibromas. The third fibroma on the left foot was located on the plantar medial aspect of the left first metatarsal–phalangeal joint. On the right foot, a single fibroma was noted on the medial aspect of the central band of the plantar fascia, measuring 2.09 cm^2^.

Sonographic examination of the left foot revealed two fibromas affixed to the medial margin of the central band of the plantar fascia, measuring 0.45 cm^2^ (#1) and 0.56 cm^2^ (#2), respectively. The fibroma plantar medial to the left first metatarsal–phalangeal joint measured 0.28 cm^2^ (#3).

Posterior tibial blocks were performed to achieve plantar anesthesia due to the size and number of lesions, utilizing 4 mL of 2% mepivacaine. The injection protocol differed from our recommendations due to the number of fibromas treated. The fibroma of the right foot was injected with a solution containing 150 IU of hyaluronidase, 0.25 mL of triamcinolone acetonide, and 0.5 mL of 0.5% bupivacaine plain. The fibromas on the left side were each injected with a total of 150 IU of hyaluronidase, 0.25 mL of triamcinolone acetonide, and 1.5 mL of 0.5% bupivacaine plain utilizing sonographic guidance.

After the first round of injections, the patient reported a 50% pain reduction and softening of all fibromas in both feet. In subsequent injections, the patient reported decreased pain and firmness of the bilateral foot fibromas and the left foot scar. The patient received a total of four injections per fibroma in both feet over a span of nine weeks. At this point, the patient was asymptomatic with a 0/10 on VAS, and injections were stopped. The fibroma on the right foot, plantar to the central band, decreased to 0.89 cm^2^. On the left foot, the two fibromas of the medial longitudinal arch decreased in size to 0.21 cm^2^ (#1) and 0.23 cm^2^ (#2), and the fibroma at the first metatarsal–phalangeal joint decreased in size to 0.12 cm^2^ (#3). At one- and two-year follow-up after injections, the patient remained asymptomatic and fibroma size did not change. The patient uses topical verapamil 15% gel twice daily for maintenance therapy.

Summary of patient demographics, dosing and treatment outcomes are referenced in Table 1.

## 4. Discussion

To our knowledge, this is the first case series to report the combined use of hyaluronidase and triamcinolone acetonide for the treatment of Ledderhose Disease. In this series, all three patients achieved pain relief and a reduction in fibroma size after three to four injections (Table 1). Injections were discontinued once patients were satisfied with their symptom relief, this explains the difference in the number of injections per patient. Our recommendation is to continue injections every 3 weeks until pain relief is achieved. This series demonstrates that doses of 150 IU hyaluronidase with a uniform dose of triamcinolone acetonide can effectively enzymatically degrade fibromas. The type of the local anesthetics used is the preference of the provider, as the addition of a local anesthetic is for immediate pain relief rather than fibroma treatment. VAS scores among all three patients prior to injections were 8/10, 9/10 and 10/10. Following completion of the entire injection series, all patients reported a 0/10 pain on VAS. At one- and two-year follow-up, patients remained pain-free, and lesion size was unchanged.

Steroids are known to reduce fibroblast proliferation and increase apoptosis rates in fibroblasts and inflammatory cells [18]. Pentland and Anderson reported that one patient experienced a decrease in fibroma size and pain after five monthly intralesional injections of 0.5–1.0 mL of triamcinolone acetonide (40 mg/mL) [19]. The patient was able to return to running activities 4 months after the last injection. Flanagan et al. also reported successful treatment of Ledderhose Disease in two cases using two triamcinolone acetonide with lidocaine administered via a fenestration technique 6 weeks apart [5]. Pain relief and fibroma size reduction were noted at 14 weeks to 6 months after initial injection. Triamcinolone acetonide injections may also help prevent recurrence, as seen in the study by Ketchum and Donahue, which reported a 50% recurrence rate after three years in patients treated with triamcinolone acetonide injections for Dupuytren’s disease [20].

Since hyaluronidase promotes extracellular matrix (ECM) breakdown and increase in tissue permeability, we hypothesize that the combination injection enhanced the effects of triamcinolone acetonide [18]. This would support the observation that combination injections are quicker and more efficient at softening and reducing pain compared to patients who receive isolated triamcinolone acetonide injections, as observed by Pentland and Anderson, and Flangan et al. [5,19]. All three patients in our study reported significant improvements in pain, firmness, and size after the second injection (6 weeks after initial injection).

The injections were overall well tolerated, with few reported complications or side effects. The most common adverse effects included mild injection site pain, burning, itching, dry skin, skin discoloration, and transient discomfort for several days post-injection. The post-injection pain associated with hyaluronidase was generally mild and manageable. In contrast, case studies on collagenase injections for Ledderhose Disease have reported significant post-injection pain and ecchymosis, often requiring protected weight-bearing for several weeks [21,22].

Surgical excision of fibromas typically yields less favorable outcomes compared to enzymatic injections, often resulting in substantial postoperative pain and high recurrence rates. Durr et al. reported recurrence rates of 100% following local excision, 80% with wide excision, and 0% with total fasciectomy combined with skin grafting [23]. Additional surgical complications include painful scarring, loss of arch height, nerve entrapment, and wound healing problems [14].

While generally safe, enzymatic injections—particularly hyaluronidase—are not without risks. Patients with a history of allergic reactions to bee stings or bovine collagen are at increased risk for hypersensitivity reactions. Allergic responses to hyaluronidase have been reported in 0.05–0.69% of cases, with angioedema and urticaria occurring in fewer than 0.1% of cases [4]. Most reactions present as erythematous edema at the injection site, particularly in patients receiving doses over 100,000 IU [4]. In our study, hyaluronidase doses were 150 IU per fibroma.

We recognize several limitations of this study. First, we used a combination of hyaluronidase and triamcinolone acetate, making it difficult to isolate the specific contribution of hyaluronidase. Although triamcinolone has demonstrated efficacy in reducing fibroma size and pain, no prior studies have evaluated the role of hyaluronidase alone in Ledderhose Disease. Future studies should investigate hyaluronidase monotherapy to better assess its independent efficacy. Following completion of injections, two out of the three patients used twice daily topical 15% verapamil gel for prevention of fibroma regrowth. We acknowledge this could have potentially impacted the effectiveness of combination injections. Further studies need to be conducted to assess the effectiveness of hyaluronidase and triamcinolone without post-injection topical verapamil use. Additionally, this is a case series, which provides a lower level of evidence. Larger, controlled studies with a control group for comparison are needed to comprehensively evaluate the utility of hyaluronidase in treating Ledderhose Disease.

## 5. Conclusions

Our case series demonstrates the use of hyaluronidase and triamcinolone acetonide injections appears safe and potentially effective for treating Ledderhose Disease in patients with both primary and recurrent fibromas. Although the current literature lacks data on hyaluronidase in this context, our results are promising. Future research should focus on larger, rigorously designed clinical trials to validate the safety, efficacy, and long-term outcomes of hyaluronidase and triamcinolone injections for Ledderhose Disease.

## Figures and Tables

**Table 1:** Summary of patient demographics, hyaluronidase dosing, and treatment outcomes in a plantar fibromatosis case series.

Case	Lesion Location	Sex	Age (yrs)	Pre-Treatment Size (cm^2^)	Post-Treatment Size (cm^2^)	Absolute Reduction (cm^2^)	Size Reduction (%)	Hyaluronidase Dose (IU/Injection)	Total Injections
1	Left midfoot	F	49	0.64	0.04	0.6	93.8	150	4
2	Left foot #1	M	59	0.23	0.11	0.12	52.2	150	3
2	Left foot #2	M	59	0.29	0.13	0.16	55.2	150	3
2	Right foot	M	59	1.51	0.89	0.62	41.1	150	3
3	Right foot	F	65	2.09	0.89	1.2	57.4	150	4
3	Left foot #1	F	65	0.45	0.21	0.24	53.3	150	4
3	Left foot #2	F	65	0.56	0.23	0.33	58.9	150	4
3	Left foot #3	F	65	0.28	0.12	0.16	57.1	150	4

## Data Availability

The original contributions presented in this study are included in the article. Further inquiries can be directed to the corresponding author.
